# Relationship between preoperative malnutrition, frailty, sarcopenia, body composition, and anthropometry in elderly patients undergoing major pancreatic and biliary surgery

**DOI:** 10.3389/fnut.2023.1135854

**Published:** 2023-02-21

**Authors:** Lijuan Wang, Pengxue Li, Yifu Hu, Bo Cheng, Lili Ding, Lei Li, Jinghai Song, Junmin Wei, Jingyong Xu

**Affiliations:** ^1^Department of Clinical Nutrition, National Center of Gerontology, Beijing Hospital, Institute of Geriatric Medicine, Chinese Academy of Medical Sciences, Beijing, China; ^2^Department of General Surgery, National Center of Gerontology, Beijing Hospital, Institute of Geriatric Medicine, Chinese Academy of Medical Sciences, Beijing, China; ^3^Department of Hepato-Bilio-Pancreatic Surgery, National Center of Gerontology, Beijing Hospital, Institute of Geriatric Medicine, Chinese Academy of Medical Sciences, Beijing, China; ^4^The Key Laboratory of Geriatrics, National Center of Gerontology, National Health Commission, Beijing Hospital, Beijing Institute of Geriatrics, Institute of Geriatric Medicine, Chinese Academy of Medical Sciences, Beijing, China

**Keywords:** malnutrition, frailty, sarcopenia, body composition, surgery

## Abstract

**Objective:**

To analyze the correlation between preoperative nutritional status, frailty, sarcopenia, body composition, and anthropometry in geriatric inpatients undergoing major pancreatic and biliary surgery.

**Methods:**

This is a cross-sectional study of the database from December 2020 to September 2022 in the department of hepatopancreatobiliary surgery, Beijing Hospital. Basal data, anthropometry, and body composition were recorded. NRS 2002, GLIM, FFP 2001, and AWGS 2019 criteria were performed. The incidence, overlap, and correlation of malnutrition, frailty, sarcopenia, and other nutrition-related variables were investigated. Group comparisons were implemented by stratification of age and malignancy. The present study adhered to the STROBE guidelines for cross-sectional study.

**Results:**

A total of 140 consecutive cases were included. The prevalence of nutritional risk, malnutrition, frailty, and sarcopenia was 70.0, 67.1, 20.7, and 36.4%, respectively. The overlaps of malnutrition with sarcopenia, malnutrition with frailty, and sarcopenia with frailty were 36.4, 19.3, and 15.0%. There is a positive correlation between every two of the four diagnostic tools, and all six *p*-values were below 0.002. Albumin, prealbumin, CC, GS, 6MTW, ASMI, and FFMI showed a significantly negative correlation with the diagnoses of the four tools. Participants with frailty or sarcopenia were significantly more likely to suffer from malnutrition than their control groups with a 5.037 and 3.267 times higher risk, respectively (for frailty, 95% CI: 1.715–14.794, *p* = 0.003 and for sarcopenia, 95% CI: 2.151–4.963, *p*<0.001). Summarizing from stratification analysis, most body composition and function variables were worsen in the ≥70 years group than in the younger group, and malignant patients tended to experience more intake reduction and weight loss than the benign group, which affected the nutrition diagnosis.

**Conclusion:**

Elderly inpatients undergoing major pancreatic and biliary surgery possessed high prevalence and overlap rates of malnutrition, frailty, and sarcopenia. Body composition and function deteriorated obviously with aging.

## 1. Introduction

The geriatric syndrome refers to a range of multifactorial health conditions representing the accumulation of multiple system impairments in older adults. Malnutrition, frailty, and sarcopenia are three common geriatric syndromes, which can substantially lead to poor outcomes, such as disability, dysfunction, falls, and perioperative complications, and thereby increase the length of hospital stay (LOS) and the cost of hospitalization, and result in long-term care or even mortality (1–5).

Malnutrition or undernutrition refers to deficiencies in nutritional intake resulting in altered body composition, and approximately 1/3 of Chinese geriatric inpatients experience malnutrition (6). In the department of hepatopancreatobiliary surgery, the prevalence of nutritional risk and malnutrition are as high as 69.7 and 56.6% in our former study (7). Frailty is characterized by a cumulative decline in the physiological capacity of multiple organ systems and increased vulnerability to endogenous and exogenous stressors, with an estimated prevalence ranging from 18.8 to 41.9% in geriatric surgical patients and from 10.4 to 37.0% in general surgical patients (8, 9). Sarcopenia is an age-related syndrome characterized by progressive and generalized loss of skeletal muscle mass and strength, which accounts for 17.4% of Chinese community-dwelling and hospitalized elderly (10). In pancreatic surgery, the prevalence is 38.8% determined by the total psoas area index in CT scan (7).

Frailty, sarcopenia, and malnutrition have independent diagnostic criteria, but share many components, such as weight loss, muscle mass, or strength loss, and often coexist or overlap in elderly inpatients (11). In a recent systematic review, it was concluded that about half of the hospitalized older patients suffer from 2 or perhaps 3 of these debilitating conditions, and standardized screening for these conditions is highly controversial to guide nutritional and physical interventions (12). Due to the significant influence of these three clinical problems on outcomes, respectively, it is important to understand the current situation and provide basal data for further cohort study. So our study aims to investigate the prevalence and overlap of these conditions in the elderly who are going to receive major pancreatic and biliary surgery.

## 2. Materials and methods

### 2.1. Participants

This study is a cross-sectional study analyzing the daily database of the Department of hepatopancreatobiliary surgery, Beijing Hospital. From December 2020 to September 2022, 205 consecutive patients undergoing major pancreatic and biliary surgery were screened, and then, 140 elderly patients were recruited in this study.

**FIGURE 1:**
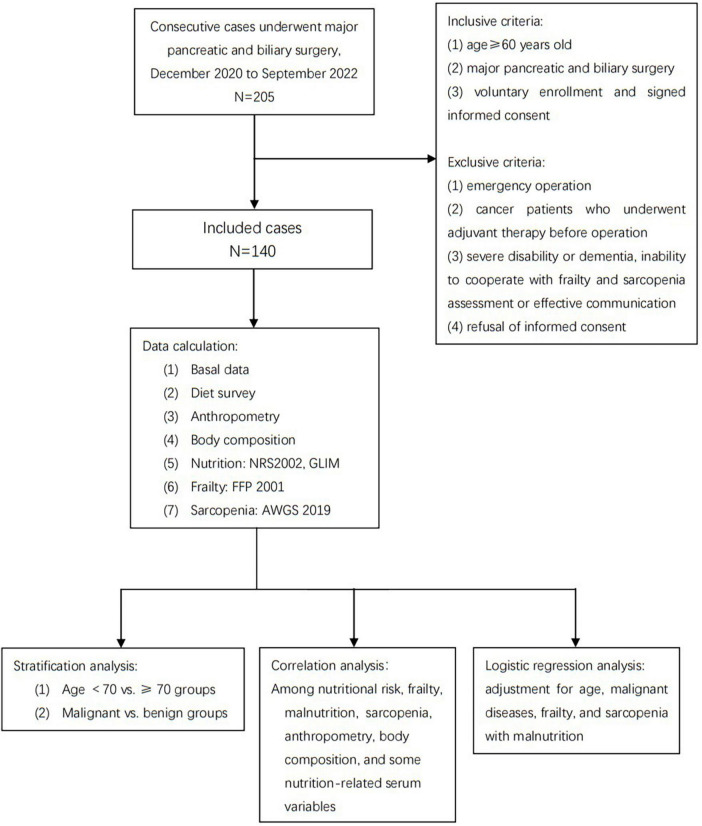
Flowchart of the study.

The inclusion criteria of this study are as follows: (1) age ≥60 years old, which is the age cut-off of older adults defined by the Nation Health Commission of China (13); (2) major pancreatic and biliary surgery, containing pancreatectomy (Whipple procedure, distal pancreatectomy, and local pancreatectomy), bile-enteral bypass due to malignant obstructive, and bile duct exploration; (3) voluntary enrollment and signed informed consent. Exclusion criteria contain (1) emergency operation; (2) cancer patients who underwent adjuvant therapy before operation; (3) severe disability or dementia, inability to cooperate with frailty and sarcopenia assessment or effective communication; (4) refusal of informed consent. The Ethics Committee of Beijing Hospital approved the study protocol and written informed consents were obtained from all participants. (Approval letter No. 2020BJYYEC-218-01). The present study adhered to the STROBE guidelines for cross-sectional study. Figure 1 shows the flowchart of this study.

### 2.2. Basal characteristics, anthropometry, and body composition

The basal data include sex, age, height, weight, body mass index (BMI), co-morbidities, and serum examination (complete blood count, liver function, renal function, albumin, glucose, et al.). According to the standard of the guidelines for prevention and control of overweight and obesity in Chinese adults, a BMI < 18.5 kg/m^2^ was defined as underweight, 18.5 kg/m^2^ ≤ BMI < 24 kg/m^2^ was normal weight, 24 kg/m^2^ ≤ BMI < 28 kg/m^2^ was considered overweight, and BMI ≥ 28 kg/m^2^ was considered obesity (14).

A diet survey was conducted after admission. We recorded the change of diet before and after the diagnosis of the original disease, and calculated the contents composition, containing protein, carbohydrate, fat, and total energy.

Anthropometry was done 1 to 2 days after admission, including calf circumference (CC) and grip strength (GS), both of which, we used the average value of the left and right sides. To assess the functional status, 15-foot and 6-meter timed walk speed (6MTW) was conducted to get the walking speed. Bioelectrical impedance analysis (BIA) was applied with the InBody 720 bioimpedance body composition analyzer (Biospace Co., Ltd., Korea). Appendicular skeletal muscle mass index (ASMI) was calculated, which was the sum of the lean muscle mass of the upper and lower extremities adjusted with height. Also, the fat-free mass index (FFMI) was recorded. Visceral fat area (VFA), waist-hip ratio (WHR), and body fat percentage (BTP) were included to reflect fat metabolism.

### 2.3. Nutritional risk screening

We used Nutritional Risk Screening 2002 (NRS 2002) for nutritional screening for each patient within 24 h after admission, which was recommended by the European Society of Parenteral Enteral Nutrition (ESPEN) (15). NRS2002 contains three aspects: nutritional impairment: weight loss, intake reduction, and lower BMI (score 0–3), the severity of disease (score 0–3), and age [(score 0–1) (< 70 years: 0 scores and ≥ 70 years: 1 score)]. Scores for the final screening take into account all these three sections range from 0 to 7 and classify patients into one of two nutritional risk stages (or groups): at low nutritional risk group (NRS 2002 score < 3), and (moderate/high) risk of malnutrition group (NRS 2002 score ≥ 3). In pancreatic surgery, an NRS2002 score of more and equal to 5 was considered at high nutritional risk with remarkable clinical meaning (16).

### 2.4. Malnutrition diagnosis and grading

The Global Leadership Initiative on Malnutrition (GLIM) criteria were implemented for malnutrition diagnosis and grading among patients with nutritional risk determined by NRS2002 (17). The framework of GLIM criteria includes three phenotypic criteria and two etiologic criteria, and the detailed items and cut-off values could be determined and modified in different centers and populations (18). In this study, we used GLIM criteria in a traditional way with the original criteria. Phenotypic criteria include (1) unintentional weight loss (WT): WT > 5% within the past 6 months, or WT > 10% beyond 6 months; (2) low BMI: BMI < 18.5 kg/m^2^ if age < 70 years, BMI < 20 kg/m^2^ if age ≥ 70 years; (3) reduced muscle mass: in our study, we used AMMI and FFMI assessed by BIA. AMMI < 7 kg/m^2^ or FFMI < 17 kg/m^2^ in men were considered patients with reduced muscle mass, and AMMI < 5.7 kg/m^2^ or FFMI < 15 kg/m^2^ in women were considered positive. Etiologic criteria include: (1) Reduced food intake: ≤50% of needs from 1 to 2 weeks, or any reduction for >2 weeks; (2) Disease burden or inflammation: in this study, most of the patients were suffering from malignancies and the co-morbidities were also taken into account. If at least one criterion was fulfilled in each section, malnutrition can be diagnosed.

The grading of malnutrition also followed the GLIM criteria. Unintentional weight loss (WT) > 10% within the past 6 months or WT > 20% beyond 6 months or low BMI (BMI < 17.0 kg/m^2^ if age < 70 years or BMI < 17.8 kg/m^2^ if age ≥ 70 years) or severe muscle deficit were defined as severe malnutrition. 5–10% Unintentional weight loss (WT) within the past 6 months or 10–20% WT beyond 6 months or low BMI (17.0 ≤ BMI < 20.0 kg/m^2^ if age < 70 years or 17.8 ≤ BMI < 22.0 kg/m^2^ if age ≥ 70 years) or Mild-to-Moderate muscle deficit were the grading criteria for moderate malnutrition.

### 2.5. Diagnosis of sarcopenia

In this study, we used the criteria for sarcopenia diagnosis recommended by the Asian Working Group for Sarcopenia (AWGS) (19). For patients in acute to chronic health care or clinical research settings, a two-step protocol was used: finding cases and diagnosis. In the first step, we tended to use objective criterion, so calf circumference (CC) (<34 cm in male, <33 cm in female) was facilitated to find cases at risk of sarcopenia, based on which, in the second step, sarcopenia can be diagnosed as follows: (1) Muscle strength: men with grip strength (GS) < 28 kg, women with GS < 18 kg; (2) Physical performance: 6-meter walk < 1 m/s; (3) AMMI: men with AMMI < 7 kg/m^2^, women with AMMI < 5.7 kg/m^2^. The result containing low ASMI and low muscle strength or low physical performance was sarcopenia, and the result containing all three criteria was severe sarcopenia.

### 2.6. Diagnosis of frailty

The Fried Frailty Phenotype (FFP) is a recommended assessment tool for frailty in geriatric patients by Chinese expert group consensus (20). FFP criteria include five physical items: (1) Shrinking: Unintentional weight loss: ≥5% of body weight in the prior year; (2) Poor endurance and energy: self-reported exhaustion; (3) Weakness: poorer GS; (4) Slowness: lower walk speed; (5) Low physical activity. Patients who fulfilled none of these five criteria were classified as the non-frailty group, who fulfilled 1 or 2 criteria were classified as the pre-frailty group, and who fulfilled ≥3 criteria were considered as the frailty group. The thresholds of GS and gait speed were referred to the AWGS criteria. Table 1 shows the comparison of all the above diagnostic tools we used in this study.

**TABLE 1:** Comparison of the four tools.

	NRS2002	GLIM	AWGS 2019	FFP 2001
Weight loss	> 5% within past 3 months(1 score) >5% within past 2 months(2 scores) >5% within past 1 months(3 scores)	WT > 5% within past 6 months WT > 10% beyond 6 months	Unintentional weight loss	Unintentional weight loss: of 10 pounds in prior year, or of 5% of body weight in prior year at follow-up
BMI	BMI < 18.5 kg/m^2^ (3 scores)	BMI < 18.5 kg/m^2^ if age < 70 years BMI < 20 kg/m^2^ if age ≥ 70 years	–	–
Muscle mass reduction	–	Reduced by validated body composition measuring techniques: FFMI by DXA or BIA, CT or MRI Anthropometric measures: calf circumferences Functional assessment: hand-grip strength	ASMI by DXA or BIA CC, SARC-F, or SARC-CalF Handgrip strength	Grip strength
Intake reduction	50–75% of normal requirement in preceding week (1 score) 25–50% of normal requirement in preceding week (2 scores) 0–25% of normal requirement in preceding week (3 scores)	≤ 50% of needs from 1 to 2 weeks any reduction for >2 weeks	–	–
Disease and inflammation burden	Patient with chronic disease, admitted to hospital due to complications. Protein requirement can be covered by oral diet or supplements (1 score) Patient confined to bed due to illness. Protein requirement can be covered by artificial feeding (2 scores) Patient in intensive care. Protein requirement is increased and cannot be covered even by artificial feeding (3 scores)	Acute disease/injury-related: Severe inflammation and mild-to-moderate inflammation Chronic disease-related: Chronic or recurrent mild-to-moderate inflammation Transient inflammation of a mild degree is excluded	Malnutrition Chronic conditions	——
Physical performance	–	–	6-meter walk time 5-time chair stand test SPPB	15-feet walk time Low physical activity level
Other	–	–	Depressive mood Cognitive impairment Repeated falls	Self-reported exhaustion

BMI, body mass index; WT, weight; FFMI, fat free mass index; DXA, Dual-energy X-ray absorptiometry; BIA, bioelectrical impedance analysis; CT, computed tomography; MRI, magnetic resonance imaging; ASMI, appendicular skeleton muscle index; CC, calf circumference; SPPB, Short Physical Performance Battery; SARC-F, Strength, Assistance walking, Rising from a chair, Climbing stairs, Falls; SARC-CalF, Strength, Assistance walking, Rising from a chair, Climbing stairs, Calf circumference, Falls.

### 2.7. Statistical analysis

The sample size was calculated by PASS software 11.0 (NCSS LLC., Kaysville, UT, USA). The confidence level was set at 0.8. According to our former study, the prevalence of malnutrition was 56.6% and we set the proportion at 60% (7). The tolerance error was set at 10%, so the two-sided confidential interval width was 0.12. The final sample size was 125. All statistical analysis was performed by IBM SPSS Statistics for Windows, version 27.0 (IBMCorp., Armonk, NY, USA). Measurement data that correspond to normal distribution were presented as mean with standard deviation (SD) and analyzed by Student’s *t*-test. Measurement data that did not correspond to normal distribution were presented as median with interquartile range (IQR) and analyzed by Mann–Whitney U test. Categorical data were presented as counts and percentages, and compared by chi-square (χ^2^) test. Correlations were analyzed by Spearman’s correlation coefficient analysis according to the classification of variables. Multivariate analysis was performed by binary logistic regression to identify potential associated factors of malnutrition. A *p*-value < 0.05 were declared as statistically significant. All figures including flowchart, overlap bubble chart, and correlation heatmap were designed and drawn by Microsoft Office (Version 2016), and the regression analysis figure was drawn by GraphPad Prism version 7.0.0 for Windows (GraphPad Software, San Diego, CA, USA).

## 3. Results

### 3.1. Basal characteristics and nutrition status

A total of 140 participants were included with a mean age of 70.0 ± 7.3 years. 58.6% (82/140) were male. 75% (105/140) of cases were malignancies, of which, 71 cases were pancreatic duct adenocarcinoma. The details of the history and blood test at admission are shown in Table 2.

**TABLE 2:** Baseline characteristics.

Variables	Basal data, *n* = 140
**Sociodemographics**	
Age, mean (SD), years	70.0 (7.3)
60–69, *n* (%)	76 (54.3)
≥ 70, *n* (%)	64 (45.7)
Male sex, *n* (%)	82 (58.6)
**Admission diagnosis**	
Malignancies, *n* (%)	105 (75.0)
Pancreatic cancer, *n* (%)	71 (50.7)
Bile duct cancer, *n* (%)	18 (12.9)
Duodenal cancer, *n* (%)	3 (2.1)
Ampulla cancer, *n* (%)	9 (6.4)
Other malignancies, *n* (%)	4 (2.9)
Benign diseases, *n* (%)	35 (25.0)
**History**	
Diabetes, *n* (%)	47 (33.6)
Chronic obstructive pulmonary disease, *n* (%)	4 (2.9)
Cardia-cerebral disease, *n* (%)	88 (62.9)
Smoking, *n* (%)	53 (37.9)
Drinking, *n* (%)	32 (22.9)
**Blood test at admission**	
White blood cell, mean (SD) × 10^9^/L	6.0 (1.7)
Hemoglobin. mean (SD) g/L	122.6 (17.3)
Platelet, mean (SD) × 10^9^/L	210.3 (61.7)
Fasting glucose, mean (SD) g/L	6.6 (2.9)
Total protein, mean (SD) g/L	64.3 (5.3)
Albumin, mean (SD) g/L	37.3 (4.3)
Pre-albumin, mean (SD) g/L	18.0 (7.7)
Alanine aminotransferase, median (IQR) U/L	21.0 (89.5)2
Creatine, mean (SD) μmoI/L	64.7 (16.5)
Triglyceride, mean (SD) mmol/L	1.5 (1.0)
Total cholesterol, mean (SD) mmol/L	4.5 (1.3)

SD, standard deviation; BMI, body mass index; IQR, interquartile range.

Table 3 shows the data for nutrition assessment. The mean BMI was 23.5 ± 3.6 kg/m^2^. 83 cases (59.3%) experienced weight loss to varying degrees, in which, 66 cases exceeded 5%. According to NRS 2002, 70.0% (*n* = 98) of cases were at risk of nutrition. 94 cases (67.1%) were malnutrition and 49 cases (35.0%) were severe malnutrition according to GLIM criteria. Based on FFP criteria, 53.6% (*n* = 75) participants were pre-frailty, and 20.7% (*n* = 29) were frailty. According to the AWGS 2019 consensus, at the step of finding cases, 52.9% (*n* = 74) cases were at risk of sarcopenia determined by reduced calf circumference, among which, in the second step, 36.4% (*n* = 31) participants were diagnosed as sarcopenia, 24.2% (*n* = 34) fulfilled the criteria of severe sarcopenia. We also reported every diagnostic criterion in each tool in Table 3 to reflect the composition of every diagnosis.

**TABLE 3:** Data of nutrition measurement.

Variables	Nutrition data, *n* = 140
**Nutrition assessment**	
BMI, mean (SD) kg/m^2^	23.5 (3.6)
BMI < 18.5 kg/m^2^, *n* (%)	8 (5.7)
18.5 ≤ BMI < 24 kg/m^2^, *n* (%)	74 (52.9)
24 ≤ BMI < 28 kg/m^2,^ *n* (%)	46 (32.9)
BMI ≥ 28 kg/m^2,^ *n* (%)	12 (8.6)
Weight at admission, mean (SD) kg	63.4 (11.0)
Weight loss, *n* (%)	83 (59.3)
Weight loss ≥ 5%, *n* (%)	66 (47.1)
Weight loss amount at admission, median (IQR) kg	3.0 (6.4)
Weight loss percentage at admission, median (IQR)%	4.3 (9.1)
NRS 2002–nutritional risk (score ≥ 3), *n* (%)	98 (70.0)
Low risk (score 3–4), *n* (%)	44 (31.4)
High risk (score 5–7), *n* (%)	54 (38.6)
**Nutrition impairment**	
Weight loss score 0/1/2/3, *n* (%)	58 (41.4)/12 (8.6)/10 (7.1)/60 (42.9)
Intake reduction score 0/1/2/3, *n* (%)	71 (50.7)/23 (16.4)/36 (25.7)/10 (7.1)
BMI score 0/3, *n* (%)	132 (94.3)/8 (5.7)
Disease burden score 0/1/2/3, *n* (%)	0 (0.0)/0 (0.0)/140 (100.0)/0 (0.0)
Age score 0/1, *n* (%)	76 (54.3)/64 (45.7)
GLIM – malnutrition, *n* (%)	94 (67.1)
Moderate-mid Malnutrition, *n* (%)	45 (32.1)
Severe malnutrition, *n* (%)	49 (35.0)
**Phenotype criteria**	
Weight loss meeting diagnostic criteria, *n* (%)	66 (47.1)
BMI meeting diagnostic criteria, *n* (%)	11 (7.9)
FFMI meeting diagnostic criteria, *n* (%)	62 (44.3)
**Etiologic criteria**	
Intake reduction meeting diagnostic criteria, *n* (%)	67 (47.9)
Disease burden meeting diagnostic criteria, *n* (%)	140 (100.0)
**FFP 2001**	
Pre-frailty, *n* (%)	75 (53.6)
Frailty, *n* (%)	29 (20.7)
Unintentional weight loss, *n* (%)	73 (52.1)
Self-reported exhaustion, *n* (%)	30 (21.4)
Low grip strengthen, *n* (%)	68 (48.6)
Low walking speed, *n* (%)	108 (77.1) [0.2pt]
Low physical activity, *n* (%)	14 (10.0)
**AWGS 2019**	
At risk of sarcopenia, *n* (%)	74 (52.9)
Low calf circumference, *n* (%)	74 (52.9)
Sarcopenia, *n* (%)	31 (36.4)
Severe sarcopenia, *n* (%)	31 (22.1)
Low grip strengthen, *n* (%)	68 (48.6)
Low walking speed, *n* (%)	108 (77.1)
Low ASMI, *n* (%)	53 (37.9)
**Diet survey**	
Energy reduction after diagnosis, median (IQR) kcal/d	76 (640.5)
Energy reduction percentage after diagnosis, median (IQR)%	5.3 (34.8)
Protein reduction after diagnosis, median (IQR) g/day	1.0 (25.2)
Protein reduction percentage after diagnosis, median (IQR)%	1.6 (46.8)
Fat reduction after diagnosis, median (IQR) g/day	0.0 (24.2)
Fat reduction after percentage after diagnosis, median (IQR)%	0.0 (49.3)
**Anthropometry**	
Calf circumference, mean (SD) cm	33.2 (3.5)
< 34 cm in male, *n* (%), *N* = 82	42 (51.2)
< 33 cm in female, *n* (%), *N* = 58	32 (55.2)
Grip strength, mean (SD) kg	24.9 (8.5)
< 28 kg in male, *n* (%), *N* = 82	50 (61.0)
< 18 kg in female, *n* (%), *N* = 58	18 (31.0)
6-meter timed walking speed, mean (SD) m/s	0.85 (0.21)
< 1 m/s, *n* (%)	108 (77.1)
**Body composition**	
Harris-Benedict equation, mean (SD) kcal/d	1279.8 (173.5)
ASMI, mean (SD) kg/m^2^	6.7 (0.9)
< 7.0 kg/m^2^ in male, *n* (%), *N* = 82	39 (47.6)
< 5.7 kg/m^2^ in female, *n* (%), *N* = 58	14 (24.1)
FFMI, mean (SD) kg/m^2^	16.5 (1.6)
< 17.0 kg/m^2^ in male, *n* (%), *N* = 82	41 (50.0)
< 15.0 kg/m^2^ in female, *n* (%), *N* = 58	21 (36.2)
Body fat percentage, mean (SD)%	27.8 (8.3)
Waist hip ratio, mean (SD)	0.92 (0.07)
Visceral fat area, mean (SD) cm^2^	83.6 (27.6)

SD, standard deviation; BMI, body mass index; IQR, interquartile range; NRS, nutritional risk screening; GLIM, global leadership initiative malnutrition; FFP, Fried frailty phenotype; AWGS, Asian Working Group for Sarcopenia; ASMI, appendicular skeleton muscle index; FFMI, fat free mass index.

**FIGURE 2:**
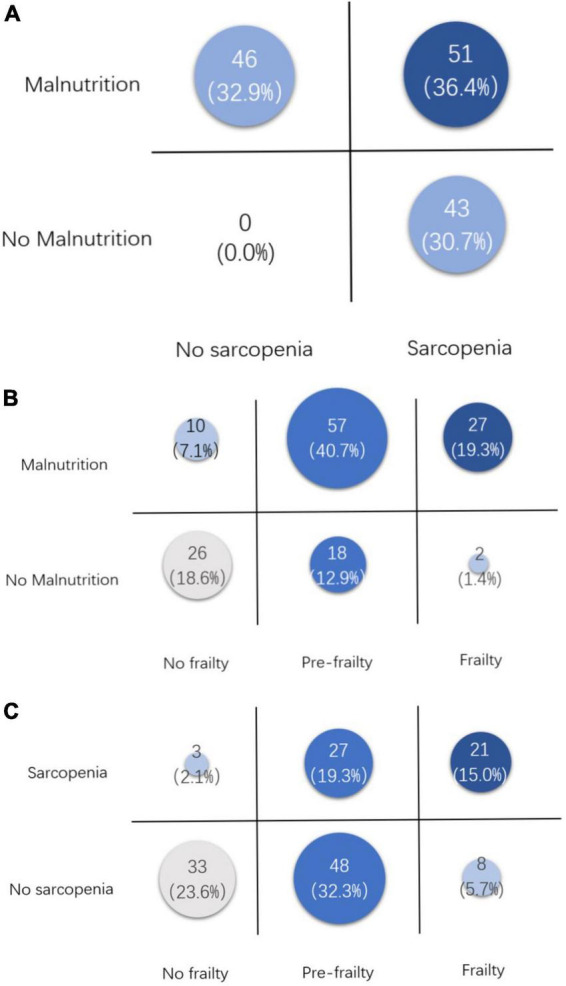
Overlaps between malnutrition, frailty, and sarcopenia. **(A)** Overlap between malnutrition and sarcopenia. **(B)** Overlap between malnutrition and frailty. **(C)** Overlap between sarcopenia and frailty.

Figure 2 displays the overlap of these three conditions, besides which, 21 (15.0%) cases fulfilled all three criteria, and 26 (18.6%) cases were considered normal by all three criteria. Furthermore, we did a stratification analysis between the patients who fulfilled three criteria and healthy patients. The results showed that there were more patients with malignant diseases (54.3% vs. 16.7%, *p* = 0,024) and older age (70.8% vs. 17.4%, *p* < 0.001) in the fulfill-three-criteria group.

A diet survey showed 71 cases had a decline in the intake of total energy, protein, and fat before and after the diagnosis of the disease. In Table 3, the amounts and percentages of reduction of energy, protein, and fat were shown in detail. Results of anthropometry and body composition analysis are also recorded in Table 3 and more men than women suffered from a decline in muscle mass and muscle-related function variables such as CC, GS, ASMI, and FFMI.

### 3.2. Stratification analysis

#### 3.2.1. Stratified by age

The patients were stratified by age and divided into the <70 years group and ≥70 years group. In Table 4, results show that the prevalence of nutritional risk, severe malnutrition, frailty, and sarcopenia were all significantly higher in the older group. Though there was no difference in the change in daily diet and weight, an obvious decline was found in both body composition and function. The changes in body composition appeared not only on the protein-related blood tests like hemoglobin, total protein, albumin, and pre-albumin, but also on the reduction of muscle mass (CC, ASMI, and FFMI), which logically affected the muscle function (e.g., GS and 6MTW).

**TABLE 4:** Stratification analysis.

Variables, *n* = 140	< 70 years	≥ 70 years	*P*	Malignant	Benign	*P*
***N***	**76**	**64**		**105**	**35**	
**Nutrition related blood test**						
Hemoglobin, mean (SD) g/L	126.1 (16.1)	118.3 (18.1)	0.001	122.2 (18.0)	124.0 (15.6)	0.629
Fasting glucose, mean (SD) g/L	6.9 (3.5)	6.2 (1.9)	0.140	6.8 (3.2)	5.7 (1.2)	0.059
Total protein, mean (SD) g/L	65.44 (4.9)	62.8 (5.5)	0.004	64.1 (5.5)	64.7 (4.7)	0.542
Albumin, mean (SD) g/L	38.6 (3.9)	35.5 (4.3)	<0.001	37.0 (4.2)	38.0 (4.6)	0.208
Pre-albumin, mean (SD) g/L	21.1 (8.0)	14.3 (5.4)	<0.001	16.6 (6.0)	21.3 (10.2)	0.008
Triglyceride, mean (SD) mmol/L	1.3 (0.7)	1.7 (1.3)	0.050	1.6 (1.0)	1.3 (1.0)	0.157
Total cholesterol, mean (SD) mmol/L	4.4 (1.1)	4.5 (1.6)	0.826	4.6 (1.4)	4.1 (0.8)	0.108
**Nutrition assessment**						
BMI, mean (SD) kg/m^2^	24.1 (3.7)	22.7 (3.4)	0.016	23.2 (3.6)	24.3 (3.6)	0.109
Weight loss, *n* (%)	42 (55.3)	41 (64.1)	0.291	70 (66.7)	13 (37.1)	0.002
Weight loss ≥ 5%, *n* (%)	35 (46.1)	31 (48.4)	0.778	56 (53.3)	10 (28.6)	0.011
Weight loss amount at admission, median (IQR) kg	3.0 (7.0)	3.0 (6.0)	0.709	3.8 (7.0)	0.0 (4.0)	0.017
Weight loss percentage at admission, median (IQR)%	4.0 (9.2)	4.7 (9.2)	0.521	5.3 (9.5)	0.0 (5.8)	0.012
NRS 2002–nutritional risk, *n* (%)	42 (55.3)	56 (87.5)	<0.001	85 (81.0)	13 (37.1)	<0.001
High risk, *n* (%)	20 (26.3)	34 (53.1)	0.001	47 (44.8)	7 (20.0)	0.009
GLIM–malnutrition, *n* (%)	47 (61.8)	47 (73.4)	0.146	75 (71.4)	19 (54.3)	0.061
Severe malnutrition, *n* (%)	18 (23.7)	31 (48.4)	0.002	41 (39.0)	8 (22.9)	0.082
FFP 2001			<0.001			0.029
Pre-frailty, *n* (%)	44 (57.9)	31 (48.4)		57 (54.3)	18 (51.4)	
Frailty, *n* (%)	6 (7.9)	23 (35.9)		26 (24.8)	3 (8.6)	
Self-reported exhaustion, *n* (%)	19 (29.7)	11 (14.5)	0.029	27 (25.7)	3 (8.6)	0.032
Low physical activity, *n* (%)	12 (18.8)	2 (2.6)	0.002	11 (10.5)	3 (8.6)	0.745
**AWGS 2019**						
At risk of sarcopenia, *n* (%)	31 (40.8)	43 (67.2)	0.002	55 (52.4)	19 (54.3)	0.845
Sarcopenia, *n* (%)	18 (23.7)	33 (51.6)	0.001	40 (38.1)	11 (31.4)	0.478
Severe sarcopenia, *n* (%)	8 (10.5)	26 (40.6)	<0.001	30 (28.6)	4 (11.4)	0.041
**Diet survey**						
Energy reduction after diagnosis, median (IQR) kcal/day	0.0 (523.0)	263.0 (817.0)	0.116	299.0 (817.0)	0.0 (0.0)	<0.001
Energy reduction percentage after diagnosis, median (IQR)%	0.0 (27.9)	14.5 (49.3)	0.074	18.7 (47.6)	0.0 (0.0)	<0.001
Protein reduction after diagnosis, median (IQR) g/d	0.0 (22.0)	10.0 (32.0)	0.105	13.0 (34.0)	0.0 (0.0)	<0.001
Protein reduction percentage after diagnosis, median (IQR)%	0.0 (32.8)	17.2 (54.1)	0.085	19.7 (51.9)	0.0 (0.0)	<0.001
Fat reduction after diagnosis, median (IQR) g/d	0.0 (12.0)	4.0 (33.0)	0.087	4.0 (28.0)	0.0 (0.0)	0.001
Fat reduction after percentage after diagnosis, median (IQR)%	0.0 (23.1)	8.9 (66.7)	0.070	8.9 (55.1)	0.0 (0.0)	0.001
**Anthropometry**						
Calf circumference, mean (SD) cm	34.3 (3.6)	32.0 (2.9)	<0.001	33.1 (3.6)	33.6 (3.3)	0.513
Grip strength, mean (SD) kg	28.0 (8.8)	21.3 (6.5)	<0.001	24.6 (8.5)	25.9 (8.5)	0.426
6-meter timed walk speed, <1 m/s, *n* (%)	0.92 (0.17)	0.74 (0.21)	<0.001	79 (75.2)	29 (82.9)	0.353
**Body composition**						
Harris-Benedict equation, mean (SD) kcal/day	1346.5 (167.4)	1199.0 (145.0)	<0.001	1273.1 (168.8)	1300.7 (188.3)	0.429
ASMI, mean (SD) kg/m^2^	6.9 (0.9)	6.4 (0.8)	0.001	6.6 (0.9)	6.8 (0.9)	0.238
FFMI, mean (SD) kg/m^2^	16.9 (1.7)	16.1 (1.3)	0.003	16.4 (1.7)	16.7 (1.5)	0.172
Body fat percentage, mean (SD)%	28.0 (8.4)	27.5 (8.2)	0.742	27.6 (8.6)	28.2 (7.6)	0.710
Waist hip ratio, mean (SD)	0.92 (0.06)	0.91 (0.07)	0.572	0.92 (0.06)	0.91 (0.07)	0.690
Visceral fat area, mean (SD) cm^2^	84.4 (28.7)	82.8 (26.5)	0.739	83.0 (27.8)	85.6 (27.5)	0.636

SD, standard deviation; BMI, body mass index; IQR, interquartile range; NRS, nutritional risk screening; GLIM, global leadership initiative malnutrition; FFP, Fried frailty phenotype; AWGS, Asian Working Group for Sarcopenia; ASMI, appendicular skeleton muscle index; FFMI, fat free mass index.

#### 3.2.2. Stratified by malignant and benign disease

When the patients were divided into malignant and benign groups, the results were completely different from the results in the groups stratified by age as above (Table 4). The main differences between malignant and benign groups were the changes in daily diet and weight, and no difference was found in body composition and function. Only prealbumin showed a significant decline in malignant disease in the benign disease group (16.6 ± 6.0 vs. 21.3 ± 10.2, *p* = 008), which was a sensitive variable to indicate recent nutrition changes. Both nutritional risk and severe nutritional risk were significantly higher in the malignant group, but no difference was found in the prevalence of GLIM-defined malnutrition. The malignant group possessed higher rates of frailty and sarcopenia.

### 3.3. Correlation between variables

**FIGURE 3:**
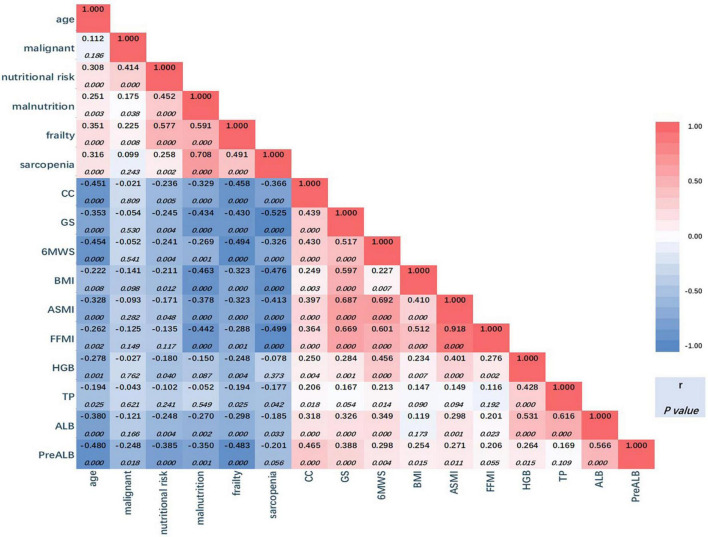
Correlation heatmap. The correlation coefficient numbers (r) are presented in the triangle, red for positive association, and blue for negative association. Darker colors indicate stronger associations (larger coefficient numbers). The significance levels for coefficients are presented below the r. CC, calf circumference; GS, grip strength; 6MWS, 6-meter walking speed; BMI, body mass index; ASMI, appendicular skeleton muscle index; FFMI, fat free mass index; HGB, hemoglobin; TP, total protein; ALB, albumin; PreALB, prealbumin.

Figure 3 is a heatmap showing the correlation between variables. From the perspective of overall color composition, the blue area shows a negative correlation between variables of serum test, body composition, and anthropometry with the four diagnostic tools. The red area could be divided into two parts: the part on the upper left of the blue area shows a positive correlation between every two of the four tools, and all six *p*-values were below 0.05; the part on the lower right of the blue area shows a positive correlation between variables of serum test, body composition, and anthropometry. In serum tests, hemoglobin, albumin, and prealbumin show a significant correlation with body composition and anthropometry. Body composition (BMI, ASMI, and FFMI) are well correlated with anthropometry (CC, GS, and 6MTW) with statistical significance.

The first column shows the correlation between age and other variables. The prevalence of nutritional risk, malnutrition, frailty, and sarcopenia were all positively correlated with age with significance, and all body composition and anthropometry variables were negatively correlated with age. Meanwhile, in the second column, nutritional risk, malnutrition, and frailty were proved to positively correlate with malignant diseases with statistical significance. However, a significant negative correlation was only found in prealbumin in all body composition and anthropometry variables (*r* = −0.248, *p* = 0.018).

### 3.4. Multivariate logistic regression analysis

**FIGURE 4:**
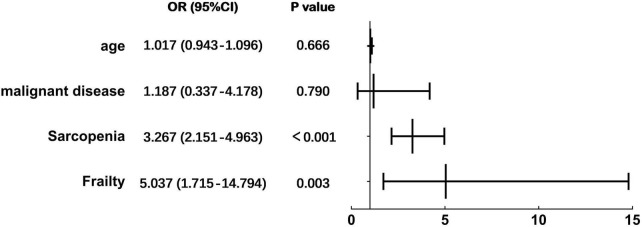
Multivariate regression analysis for malnutrition.

After adjustment for age, malignant diseases, frailty, and sarcopenia with malnutrition as the dependent variables, multivariate logistic regression analysis showed that participants with frailty or sarcopenia were significantly more likely to suffer from malnutrition than their control groups with a 5.037 and 3.267 times higher risk, respectively (for frailty, 95% CI: 1.715–14.794, *p* = 0.003 and for sarcopenia, 95% CI: 2.151–4.963, *p* < 0.001) (Figure 4).

## 4. Discussion

With increasing global aging problems, aging-related debilitating disorders, so-called geriatric syndrome, are becoming the hotspots of geriatric research. All frailty, sarcopenia, and malnutrition are components of geriatric syndrome and are closely interrelated and interdependent. Surgical patients suffer from a double attack of disease and aging. In our study, the prevalence of nutritional risk, and malnutrition are 70.0 and 67.1%, respectively, which are higher than in elderly patients with other gastrointestinal diseases (21). The prevalence of frailty is 20.7%, which is familiar to former articles and at a relatively higher proportion (9). The prevalence of sarcopenia is 36.4%, which is nearly the same as our data collected in pancreatic surgery diagnosed by a CT scan (7).

Diagnosis of malnutrition, frailty, and sarcopenia depend on the diagnostic tools, different tools might lead to different prevalence (22). In a recent systemic review, 18 tools of frailty diagnosis were reported, in which, FFP was the most commonly used one. Meanwhile, EWGSOP (European Working Group on Sarcopenia in Older People) criteria was the most commonly used in all and AWGS was the most commonly implemented in Asia. And for malnutrition, about thirteen tools were mentioned, besides which, BMI only and BMI with albumin were considered to be diagnostic criteria in three articles (12). However, it is difficult to avoid bias when calculating overlap data between different tools and it is still a controversy in this field. So in our study, we chose FFP, WGS, NRS2002, and GLIM to avoid selection bias.

Table 1 displays the comparison of FFP, WGS, NRS2002, and GLIM, which reflect the commonality and individuality of the tools. Weight loss was the only criterion shared by the four tools, which is not only for nutrition assessment but also a sensitive precursor for tumor diagnosis, especially for pancreatic cancer (23). Besides weight loss, NRS2002 and GLIM contain age, BMI, intake reduction, and assessment of disease (inflammation burden), which are relatively more comprehensive to assess the nutrition status. But no muscle assessment was contained in NRS2002, and GLIM contains the evaluation of muscle, but with a large range of measuring techniques. AWGS2019 and FFP2001 criteria are based on muscle assessment. The overlaps between frailty or sarcopenia and malnutrition were 19.3 and 36.4%, which are higher than was reported before (12). AWFS2019 criteria focus on both muscle mass and muscle function to diagnose sarcopenia, meanwhile, FFP2001 criteria only focus on muscle function and function-related symptoms like cognitive and behavioral impairment. So the overlap of sarcopenia and frailty (15.0%) was not as large as expected. Moreover, in this study, 21 (15.0%) cases fulfilled all three criteria, and 26 (18.6%) cases were considered normal or no risk by all three criteria. Therefore, due to different clinical values and low overlap rates, these diagnostic criteria would still coexist, and more comprehensive tools may be created and validated in the future.

According to the guidelines of ESPEN, malnutrition, sarcopenia, and frailty were treated as parallel definitions (24). The links between malnutrition and sarcopenia or frailty have already been explored in several cross-sectional studies, especially in older patients with chronic disease (25–27). In our study, in surgical patients, the correlations between these conditions were proved to be statistically significant, which were shown in Figure 3. However, it is difficult to judge the causal relationship between any two of these three statuses. Theoretically speaking, in this population, original surgical diseases lead to intake reduction and weight loss, which affected nutrients digestion and absorption, and then gave rise to the change in body composition, especially the change of muscle mass, sequentially muscular dysfunction. Nutrition risk or malnutrition seems to be the initiating factor (28, 29). Our results indicated that sarcopenia and frailty seemed to be risk factors for malnutrition, however, longitudinal studies are needed.

From the perspective of body composition in the criteria, BMI may not be sensitive enough to be used in the surgical population, only 5.7% of cases were lower than 18.5 kg/m^2^, and nearly 40% of patients suffered from overweight and obesity, in which, nearly 20% were sarcopenia (7). Even in pancreatic surgery, higher BMI was treated as a risk factor for a fatty pancreas and postoperative pancreatic fistula rather than a nutrition parameter (30). FFMI and ASMI, which reflect the real change in muscle mass, had become the focus of diagnostic criteria. In this study, FFMI accounted for 44.3% of phenotype criteria in GLIM, second only to weight loss. And it was proved to be well consistent in GLIM-defined malnutrition in this study and other reports (31). ASMI was the sum of the lean muscle mass of the upper and lower extremities adjusted with height, which was reported to be used in GLIM and well related to sarcopenia and frailty (32, 33). So with the improvement of availability and simplification of the examination method, ASMI and FFMI will become more popular in clinical practice.

In this study, we did stratification analyses by age and malignancy. Like reports from other centers, it was no doubt that nutritional status became worse with aging and malignant diagnosis (34). However, from Table 4, by comparing the data from the two stratifications, an interesting phenomenon was notable. In the age stratification, the significant differences were mainly in the changes in body composition and its related parameters, including basic metabolic rate (Harris-Benedict equation), muscle mass (CC, ASMI, and FFMI), muscle function (GS and 6MWS), BMI, and serum test (hemoglobin, total protein, albumin, and prealbumin), all of which reflected the long-term changes of the body due to aging rather than disease. Meanwhile, in the stratification of malignant diseases, the significant differences between malignant and benign groups were only weight loss and intake reduction, which were short-term changes due to the pathophysiologic characteristics of cancer, but no change in body composition existed. In the serum test, only pre-albumin was significantly lower in the malignant group, which has been proposed to be a useful nutritional biomarker due to its shorter half-life than albumin and correlated with different nutritional markers and higher mortality risk (35). So when referring to preoperative therapy, for patients with advanced age, we must pay attention to both nutrition support and function exercise, to improve long-term nutrition and function problems caused by aging, and increase preoperative reservation, which was defined as “prehabilitation” and needed a relatively longer period (36). And for cancer patients with nutritional risk or malnutrition, we should commit to increasing intake and improving nutrition status by different support routes even for a short period (16, 37). Prealbumin might be a biomarker to monitor the effectiveness of preoperative nutrition support but needs further study.

As we know, few researchers have been reported to study the effect of two of the three conditions in older adults, but the three conditions are rarely studied together (38). This study is the first one to study the overlap of these three conditions in pancreatic and biliary surgery. Since this is a cross-sectional study, we tried our best to follow the STROBE statement, but there must be some limitations that are difficult to avoid. First, we used a relatively lower confidence level (0.8) and prevalence of malnutrition to determine the sample size, which may underestimate the sample size, especially when we did the stratification analysis; second, a cross-sectional study could not verify the causal relationship. Although we used multivariate regression analysis, the aim was to explore the possible relevance and provide the necessary direction for future cohort studies. Third, the sample population is elderly, so whether the tangent point value can represent other populations should be a deeper study field and need more work.

## 5. Conclusion

Elderly inpatients undergoing major pancreatic and biliary surgery had a high prevalence and overlap rates of malnutrition, frailty, and sarcopenia. Body composition and function deteriorated obviously with aging. Patients with malignant diseases often suffer from short-term nutrition changes like intake reduction. Simple and effective biomarker needs to be explored and validated. Rational preoperative prehabilitation containing nutrition support and exercise should be considered in this population to reduce postoperative complications and mortality.

## Data availability statement

The data supporting this study’s findings are available from the corresponding author upon reasonable request. Requests to access the datasets should be directed to JX, xujingyong@bjhmoh.cn.

## Ethics statement

The studies involving human participants were reviewed and approved by the Ethics Committee of Beijing Hospital. The patients/participants provided their written informed consent to participate in this study.

## Author contributions

JX and JW: conception, design, and administrative support. JX, LL, JS, and JW: provision of study materials or patients. JX, YH, PL, LW, LD, and BC: collection, assembly of data, data analysis, and interpretation. LW, YH, and JX: manuscript writing. LW, PL, YH, BC, LD, LL, JS, JW, and JX: final approval of manuscript. All authors contributed to the article and approved the submitted version.
